# Long noncoding RNA LINC00941 promotes pancreatic cancer progression by competitively binding miR-335-5p to regulate ROCK1-mediated LIMK1/Cofilin-1 signaling

**DOI:** 10.1038/s41419-020-03316-w

**Published:** 2021-01-04

**Authors:** Jie Wang, Zhiwei He, Jian Xu, Peng Chen, Jianxin Jiang

**Affiliations:** 1 Department of Hepatobiliary Surgery, Renmin Hospital of Wuhan University, Wuhan, Hubei China; 2 Department of Hepatic-Biliary-Pancreatic Surgery, The Affiliated Hospital of Guizhou Medical University, Guiyang, Guizhou China

**Keywords:** Pancreatic cancer, Long non-coding RNAs

## Abstract

An accumulation of evidence indicates that long noncoding RNAs are involved in the tumorigenesis and progression of pancreatic cancer (PC). In this study, we investigated the functions and molecular mechanism of action of LINC00941 in PC. Quantitative PCR was used to examine the expression of LINC00941 and miR-335-5p in PC tissues and cell lines, and to investigate the correlation between LINC00941 expression and clinicopathological features. Plasmid vectors or lentiviruses were used to manipulate the expression of LINC00941, miR-335-5p, and ROCK1 in PC cell lines. Gain or loss-of-function assays and mechanistic assays were employed to verify the roles of LINC00941, miR-335-5p, and ROCK1 in PC cell growth and metastasis, both in vivo and in vitro. LINC00941 and ROCK1 were found to be highly expressed in PC, while miR-335-5p exhibited low expression. High LINC00941 expression was strongly associated with larger tumor size, lymph node metastasis, and poor prognosis. Functional experiments revealed that LINC00941 silencing significantly suppressed PC cell growth, metastasis and epithelial–mesenchymal transition. LINC00941 functioned as a molecular sponge for miR-335-5p, and a competitive endogenous RNA (ceRNA) for ROCK1, promoting ROCK1 upregulation, and LIMK1/Cofilin-1 pathway activation. Our observations lead us to conclude that LINC00941 functions as an oncogene in PC progression, behaving as a ceRNA for miR-335-5p binding. LINC00941 may therefore have potential utility as a diagnostic and treatment target in this disease.

## Introduction

Pancreatic cancer (PC) is one of the most common malignancies of the digestive system with a 5-year survival rate of <10%. PC is therefore a major cause of cancer-related morbidity and mortality worldwide^1^. Early loss of symptoms, rapid disease progression, and lack of effective treatment all contribute to the poor prognosis of patients with this disease^2^. Although neoadjuvant chemotherapy and targeted drugs have improved the management of PC, surgical resection remains the most effective option^3^. Unfortunately, PC is commonly asymptomatic during the earliest stages of the disease and progresses rapidly such that the majority of patients are diagnosed at an advanced stage, where radical resection is no longer an option^3^. Therefore, there is a significant unmet need not only for new drug targets, but also for sensitive biomarkers that can identify PC at an early stage, enabling earlier and more intensive treatments that can improve the current dismal prognosis for patients with this disease.

Long noncoding RNAs (lncRNAs) are functional gene transcripts of >200 nucleotides in length that lack the ability to encode protein directly^4^. lncRNAs have been reported to regulate a variety of biological processes, such as the cell cycle, growth, angiogenesis, metastasis, apoptosis, transcriptional modification, and drug resistance^5^. An accumulation of evidence indicates that dysregulation of lncRNAs is strongly associated with the pathogenesis of cancer, a finding that can be attributed to their oncogenic or tumor suppressive properties. Previous studies have suggested that the lncRNA LINC01111 suppresses PC aggressiveness through its ability to competitively bind miR-3924 to upregulate DUSP1, which inhibits SAPK/JNK phosphorylation^6^. Conversely, a study by Lei et al. has shown that the lncRNA LINC00976 promotes PC progression by behaving as a competitive endogenous RNA (ceRNA), acting as a molecular sponge for miR-137 (ref. ^7^). This prevents miR-137 from binding and suppressing OTUD7B mRNA, resulting in OTUD7B-dependent activation of the EFGR/MAPK pathway. The modulatory impacts of the lncRNA LINC00941 have been examined in gastric cancer, where high expression of this RNA was shown to be associated with cell proliferation and metastasis^8^. However, the function and mechanism of action of LINC00941 in PC remain unknown.

MiRNAs are small noncoding RNAs of ~23 nucleotides in length that play a regulatory role in cells by directly interacting with the 3′-untranslated region (UTR) of target mRNAs, resulting in mRNA degradation or posttranscriptional inhibition^9^. Increasing evidence shows that abnormal miRNA expression is significantly associated with tumorigenesis and tumor progression, including in PC^10^. At the molecular level, lncRNAs indirectly regulate gene expression through their ability to function as miRNA molecular “sponges”, competitively binding and sequestering miRNAs, thereby antagonizing the miRNA-mediated suppression of target mRNAs^11,12^.

In this study, we employed techniques to manipulate lncRNA expression to investigate the role and underlying mechanism of action of LINC00941 in the pathogenesis of PC. We assessed the expression of LINC00941 in 54 pairs of PC and adjacent normal tissues, and identified for the first time a correlation between LINC00941 expression and clinicopathological characteristics and patient prognosis. Furthermore, we show that LINC00941 acts as an oncogenic RNA to promoted cell growth and metastasis by functioning as an miRNA sponge for miR-335-5p, which otherwise targets and suppresses ROCK1 expression to inhibit downstream LIMK1/Cofilin-1 activity. The LINC00941/miR-335-5p/ROCK1 signaling axis may therefore present a novel target for the development of diagnostic biomarkers and therapeutics for the treatment of PC.

## Materials and methods

### Patient samples

Fifty-four tumor tissues and paired adjacent normal tissues were obtained from patients who underwent surgical resection for PC at the Renmin Hospital of Wuhan University from 2018 to 2020. After collection, all tissue samples were immediately stored at −80 °C. The clinicopathological characteristics of patients are summarized in Table 1. This study was approved by the Ethics Committee of Renmin Hospital of Wuhan University.

**Table 1:** General clinicopathological characteristics of patients.

Clinical epidemiology and clinicopathologic feature	LINC00941		*p* Value
	Low expression	High expression	
All cases	22	32	
Age			
≤50	10	13	0.7841
>50	12	19	
Gender			
Male	14	17	0.5772
Female	8	15	
Diameter of tumor			
≤2	14	10	**0.0266**
>2	8	22	
Pathological grading			
I/II	12	19	0.7841
III/IV	10	13	
Lymphatic metastasis			
Negative	16	12	**0.0141**
Positive	6	20	
Distant metastasis			
Negative	17	23	0.7582
Positive	5	9	
TNM stage			
I/II	15	19	0.5756
III/IV	7	13	

Bold means *p* value is less than 0.05.

### Cell culture and transfection

The human PC cell lines AsPC-1, BxPC-3, PANC-1, MIA PaCa-2, and Capan-2, and the human normal pancreatic ductal epithelial cell line (HPDE) were purchased from the American Type Culture Collection. HPDE, AsPC-1, and BxPC-3 cells were cultured in RPMI 1640 medium (Hyclone, USA) supplemented with 10% FBS (Gibco, USA). The remaining cell lines were cultured in DMEM medium (Hyclone). All cultures were maintained at 37 °C in a humidified atmosphere of 5% CO_2_.

Vectors encoding ROCK1 (Rock1 vector), negative control (NC vector), ROCK1 siRNA (si Rock1), negative control ROCK1 siRNA (si Rock1 NC), miRNA mimic (miR-335-5p mimic), control mimic, miRNA inhibitor (miR-335-5p inhibitor), and control inhibitor were purchased from Ribobio (Guangzhou, China). Lentiviruses for LINC00941 silencing (sh-Linc00941-1 and sh-Linc00941-2), for the silencing control (sh-NC), for LINC00941 overexpression (oe-Linc00941) and the overexpression control (oe-NC) were purchased from Genechem (Shanghai, China). Cell transfections and infections were carried out according to the manufacturer’s protocol.

### Real-time quantitative polymerase chain reaction

TRIzol reagent (Invitrogen, USA) was used to extract total RNA from PC tissues or cells according to the manufacturer’s protocol. The ReverTra Ace qPCR RT Kit (Takara, China) was used to reverse transcribe total RNA into cDNA. The SYBR Green Real-time PCR Master Mix (Takara) was used for qPCR with cDNA as the template and GAPDH or U6 used as internal controls. The relative expression of target genes was calculated and analyzed using the 2^−ΔΔCt^ method. All PCR primer sequences used in this study are detailed in Supplemental Table 1.

### Dual-luciferase reporter gene essay

Bioinformatics methods were used to predict and analyze the target gene of miR-335-5p. Wild type (WT) and mutant (MUT) sequences for LINC00941 and ROCK1 were cloned into the pGL3 dual-luciferase reporter vector. Lipofectamine 2000 (Invitrogen, USA) was used for all co-transfection assays. For miR-335-5p overexpression assays, PANC-1 cells were co-transfected with expression plasmids and either miR-335-5p mimic or control mimic. In miR-335-5p inhibitory assays, PANC-1 cells were co-transfected with expression plasmids and either miR-335-5p inhibitor or control inhibitor. At 48 h post transfection, the relative luciferase activity was determined using the Dual-Luciferase Reporter kit (Promega, USA) in accordance with the manufacturer’s instructions.

### RNA immunoprecipitation assay

The Magna RNA immunoprecipitation (RIP) assay kit (Millipore, USA) was used to investigate interactions between experimental targets in accordance with the manufacturer’s instructions. PC cells were treated with RIP lysis buffer prior to the addition of magnetic beads conjugated with Ago2 or control IgG antibodies (Sigma-Aldrich). RT-qPCR was subsequently performed to determine the levels of LINC00941 and miR-335-5p RNA in the immunoprecipitates.

### Transwell assay

Cell migration and invasion was evaluated using transwell migration chambers (24-well chambers; BD biosciences, USA). For cell invasion studies, the chamber inserts were precoated with Matrigel (1:8 ratio in DMEM). For both assays, 1 × 10^4^ cells in 200 μl of serum-free medium were seeded into the upper chamber of each well. The lower chambers were filled with 800 µl DMEM supplemented with 20% fetal bovine serum. The cells were then cultured for 24–30 h. Subsequently, the upper chambers were washed with phosphate-buffered saline (PBS), fixed with 4% paraformaldehyde (PFA), and stained with 1% crystal violet. An Olympus BX51 microscope was used to photograph the cell layers.

### Cell proliferation assay

Transfected PC cells were cultured in 96-well plates (1 × 10^4^ cells per well). To evaluate proliferation, each well was supplemented with 10 µl CCK-8 solution (Dojindo, Japan) and the 96-well plate then returned to the incubator for 1 h. A microplate reader was then used to measure the absorbance at 450 nm.

### Colony formation assay

Transfected PC cells were cultured in six-well plates (1 × 10^4^ cells per well) for 1–2 weeks, with medium changes every 2 days. Subsequently, the six-well plates were washed with PBS, fixed with 4% PFA, stained with crystal violet, and finally photographed by microscope.

### Western blotting analysis

Cell lysates of equal protein concentration were separated by 10% sodium dodecyl sulfate–polyacrylamide gel electrophoresis and then transferred to polyvinylidene fluoride (PVDF) membranes. The PVDF membranes were blocked with skimmed milk (5%) and then incubated with primary antibody against specific target proteins of interest. Antibodies against ROCK1, LIMK1, Cofilin-1, and p-Cofilin-1 were purchased from Cell Signaling Technology (CST, Danvers, MA, USA) and were used at a dilution of 1:500. Antibodies against N-cadherin, E-cadherin, Vimentin, and GAPDH were purchased from Abcam (Cambridge, MA, USA) and were all used at a dilution of 1:1000. Subsequently, membranes were washed and incubated with horseradish peroxidase (HRP)-conjugated secondary antibodies. Finally, the EasyBlot ECL kit (Sangon, China) was used to visualize the protein bands.

### In vivo tumorigenicity and metastasis mouse models

A subcutaneous transplantation model was employed to evaluate tumorigenicity in vivo. Transfected cells were prepared in suspension (1 × 10^6^ cells in 100 μl PBS) and then injected subcutaneously into the right armpit of female mice at 4 weeks. Mouse weight and subcutaneous tumor volume were recorded weekly. After 8 weeks of feeding, mice were culled and tumor tissues then harvested and processed for immunohistochemical analysis.

A metastasis model was also established to examine the metastatic potential of tumor cells in vivo. Transfected cells were prepared in suspension (1 × 10^6^ cells in 100 μl PBS) and then injected into the tail vein of anesthetized mice. After death or 10 weeks of feeding, the mice were culled, and the liver and lungs then removed by careful dissection. Finally, the number of metastases were counted by macroscopic and microscopic analytical methods.

These studies were approved by the Animal Research Ethics Committees at Renmin Hospital of Wuhan University.

### Immunohistochemistry

Mouse tumor tissues were embedded in paraffin blocks and cut into 4-μm thick sections. Tissue sections were incubated with Ki67 and PCNA primary antibodies, and then HRP-conjugated secondary antibody. 3,3′-diaminobenzidine was used as chromogen to visualize Ki67- and PCNA-positive staining.

### Immunofluorescence assays

PANC-1 cells were fixed with 4% PFA and incubated with 5% goat serum, 3% BSA, and 0.1% Triton-X100. The cells were then incubated with primary antibodies against filamentous actin (F-actin) and β-tubulin (1:100 dilution; Abcam) at 4 °C for 20 h. Then cells were then incubated with CY3-conjugated goat anti-rabbit and FITC-conjugated goat anti-mouse secondary antibodies, at 37 °C for 1 h. Cell nuclei were stained with DAPI (CST). The cells were then imaged using a laser scanning confocal microscope.

### RNA fluorescence in situ hybridization

Cultured PC cells were fixed with 4% PFA at 37 °C for 30 min. The cells were then incubated with the fluorescence in situ hybridization (FISH) probe in the dark in accordance with the manufacturer’s instructions (Ribobio, Guangzhou, China). After washing the cells two times with PBS/0.5% Tween-20 (PBST), cell nuclei were stained with DAPI. After two subsequent washes with PBST, the cells were blocked with glycerin. The cells were imaged using a laser scanning confocal microscope.

### Bioinformatic analysis

The Gene Expression Profiling Interactive Analysis (GEPIA) website was used for analyzing the gene expression based on the TCGA database. We searched the LINC00941 expression in the “Expression DIY boxplot”, and the survival analysis was used for estimating the “survival plots” in the website. The raw data of gene expression profiling were available in GEO database (GSE63124). The bioinformatic analysis software was used to detect the LINC00941 expression in the different group patient specimens. To identify the target of LINC00941, the Starbase3.0 (http://starbase.sysu.edu.cn), seedVicious (https://seedvicious.essex.ac.uk), and LncBase (http://carolina.imis.athena-innovation.gr) were performed to screen the potential binding sites, then obtained the potential miRNAs by Venn diagrams (http://bioinformatics.psb.ugent.be/webtools/Venn/).

### Statistical analysis

All data presented are from at least three experiments and are presented as the mean ± standard deviation. Statistical analysis was performed using the two-tailed Student’s *t* test or one-way analysis of variance. The correlation between lncRNA LINC00941 expression and clinicopathological features was assessed via Fisher’s exact test or Kruskal–Wallis test. SPSS 21.0 software was used for all statistical analysis. *P* < 0.05 was considered statistically significant.

## Results

### LINC00941 is highly expressed in PC tissues and cell lines, and is associated with metastasis, tumor size, and poor prognosis

**Fig. 1:**
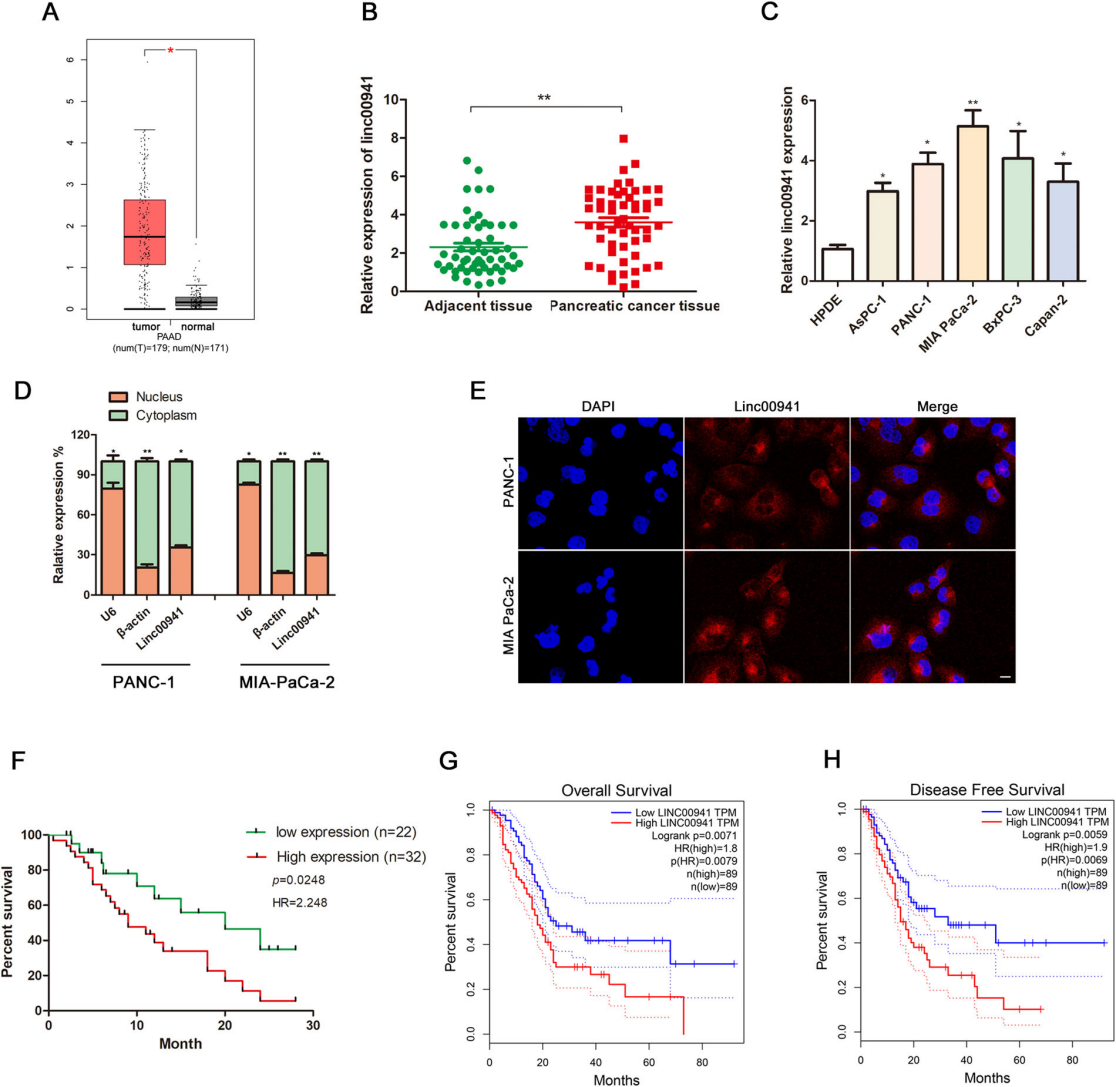
LINC00941 was highly expressed in PC tissues and cells and predicted poor prognosis. **A** The expression of LINC00941 in PC tissues obtained from TCGA database. **B** RT-qPCR analysis of LINC00941 expression in PC tissues. **C** A differential expression pattern of LINC00941 was observed in PC cell lines. **D** The relative expression of LINC00941 in nucleus and cytoplasm. **E** RNA-FISH localization of LINC00941 in PC cells. **F** The survival curve of different expression of LINC00941 in PC. **G** The overall survival months in patients with PC obtained from TCGA database, the dashed curves represented 95% confidence interval. **H** The disease-free survival months in patients with PC obtained from TCGA database, the dashed curves represented 95% confidence interval. All experiments were performed three times and data were presented as mean ± SD. *p < 0.05, **p < 0.01, ***p < 0.001.

The GEPIA network database (http://gepia.cancer-pku.cn/) was used to investigate whether LINC00941 was differentially expressed in PC (Fig. 1A), while the GEO database also confirmed that LINC00941 was highly expressed in PC tissues (Supplemental Fig. 1A). LINC00941 expression in 54 pairs of cancerous and noncancerous adjacent patient tissues was then examined by RT-qPCR to verify the findings from our initial database analyses. The RT-qPCR analysis indicated that LINC00941 RNA expression was significantly elevated in cancerous tissues, when compared with adjacent normal tissues (Fig. 1B). Furthermore, LINC00941 RNA was overexpressed in PC cell lines, when compared with HPDE cells (Fig. 1C). To determine the localization of LINC00941 in PC cells, LINC00941-specific PCR was performed using RNA purified from the cytoplasm and nucleus. Analysis of these subcellular fractions revealed that LINC00941 RNA was mainly concentrated in the cytoplasm of PC cells, a finding that was further supported by the results of our FISH analysis (Fig. 1D, E).

To categorize PC patients according to high or low LINC00941 expression, we performed ROC curve analysis of the LINC00941 expression data and calculate the cutoff value. The Youden index was 0.408, the corresponding cutoff value was 3.016, and the area under curve was 0.755 (Supplemental Fig. 1B, C). Using the calculated cutoff value, 32 patients were assigned to the high-expression group and 22 patients to the low-expression group. The correlation between clinicopathological characteristics and LINC00941 expression is shown in Table 1. The results revealed that high expression of LINC00941 strongly correlated with larger tumor size and lymph node metastasis. Notably, Kaplan–Meier analysis of overall survival using patient follow-up information revealed that high LINC00941 expression was related to poor patient prognosis (Fig. 1F). Data from the TCGA database further supported our findings (Fig. 1G, H).

### LINC00941 promotes cell proliferation, migration, invasion, and epithelial–mesenchymal transition in vitro

**Fig. 2:**
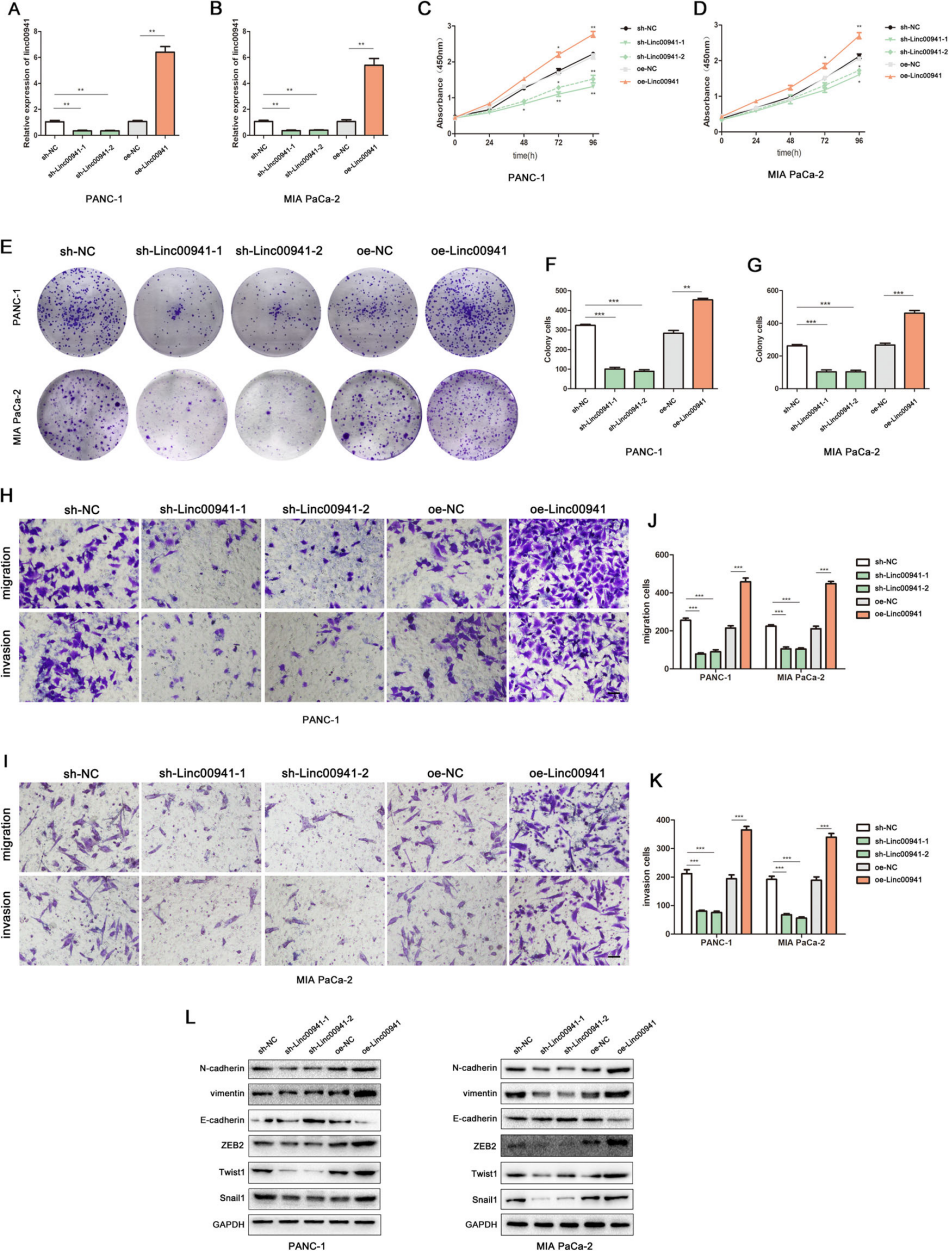
LINC00941 promoted cell proliferation, migration, invasion, and EMT in vitro. **A**, **B** The effects of transfection of lentiviral transduction in PC cell lines were detected by PCR assay. **C**, **D** CCK-8 assay was performed to test the cell viability and proliferation in upregulated and downregulated LINC00941 groups. **E**–**G** Colony formation assay was performed to test the cell colony ability in upregulated and downregulated LINC00941 groups. **H**–**K** Transwell assay was performed to analyze the migrated and invaded abilities in PC cell lines. **L** The protein expressions of N-cadherin, E-cadherin, Vimentin, ZEB2, Twist1, and Snail1 were detected in upregulated and downregulated LINC00941 groups by western blotting. *p < 0.05, **p < 0.01, ***p < 0.001.

To assess the effects of LINC00941 expression on PC cell proliferation and metastatic potential, MIA PaCa-2, and PANC-1 cells were either transduced with lentivirus encoding short interfering RNAs for LINC00941 suppression, or transduced with lentivirus encoding LINC00941 cassettes for LINC00941 overexpression. The effects of lentiviral transduction were significant, when compared with those of the controls (Fig. 2A, B). The CCK-8 assay was used to investigate the viability of PC cells, in which LINC00941 had either been suppressed or overexpressed following lentiviral transduction. These assays revealed that suppression of LINC00941 expression significantly suppressed cell viability, while elevated LINC00941 expression enhanced cell viability (Fig. 2C, D). In addition, colony formation assays revealed that LINC00941 silencing suppressed colony formation, while elevated LINC00941 expression enhanced colony formation, when compared with controls (Fig. 2E–G). To evaluate the effects of LINC00941 expression on the metastatic potential of PC cells, transwell assay was performed. These assays revealed that LINC00941 silencing significantly reduced the migration and invasion of PC cells, when compared with the controls. Conversely, overexpression of LINC00941 significantly enhanced cell migration and invasion (Fig. 2H–K). Expression of the proteins N-cadherin, Vimentin, and E-cadherin is closely associated with the epithelial–mesenchymal transition (EMT), a key biological process that epithelial-derived malignant tumor cells undergo to enable them to migrate and invade^13^. Western blotting was performed to detect the expression of these EMT-related proteins in PC cells following the lentiviral-mediated upregulation or downregulation of LINC00941 expression. Interestingly, the results indicated that elevated LINC00941 levels promoted N-cadherin, ZEB2,Snail1, Twist1, and Vimentin protein expression and inhibited E-cadherin protein expression in these cells, suggesting the transition from an epithelial to mesenchymal phenotype. Conversely, LINC00941 knockdown suppressed the ability of PC cells to acquire the EMT phenotype (Fig. 2L). And the RT-qPCR results indicated that LINC00941 significantly promoted N-cadherin, ZEB2, Snail1, Twist1, and Vimentin mRNA, and inhibited E-cadherin expression, while the LINC00941 knockdown inhibited the EMT marker expression, and elevated E-cadherin mRNA (Supplemental Fig. 2A, B).

### Suppression of LINC00941 expression inhibits PC cell proliferation and metastasis in vivo

**Fig. 3:**
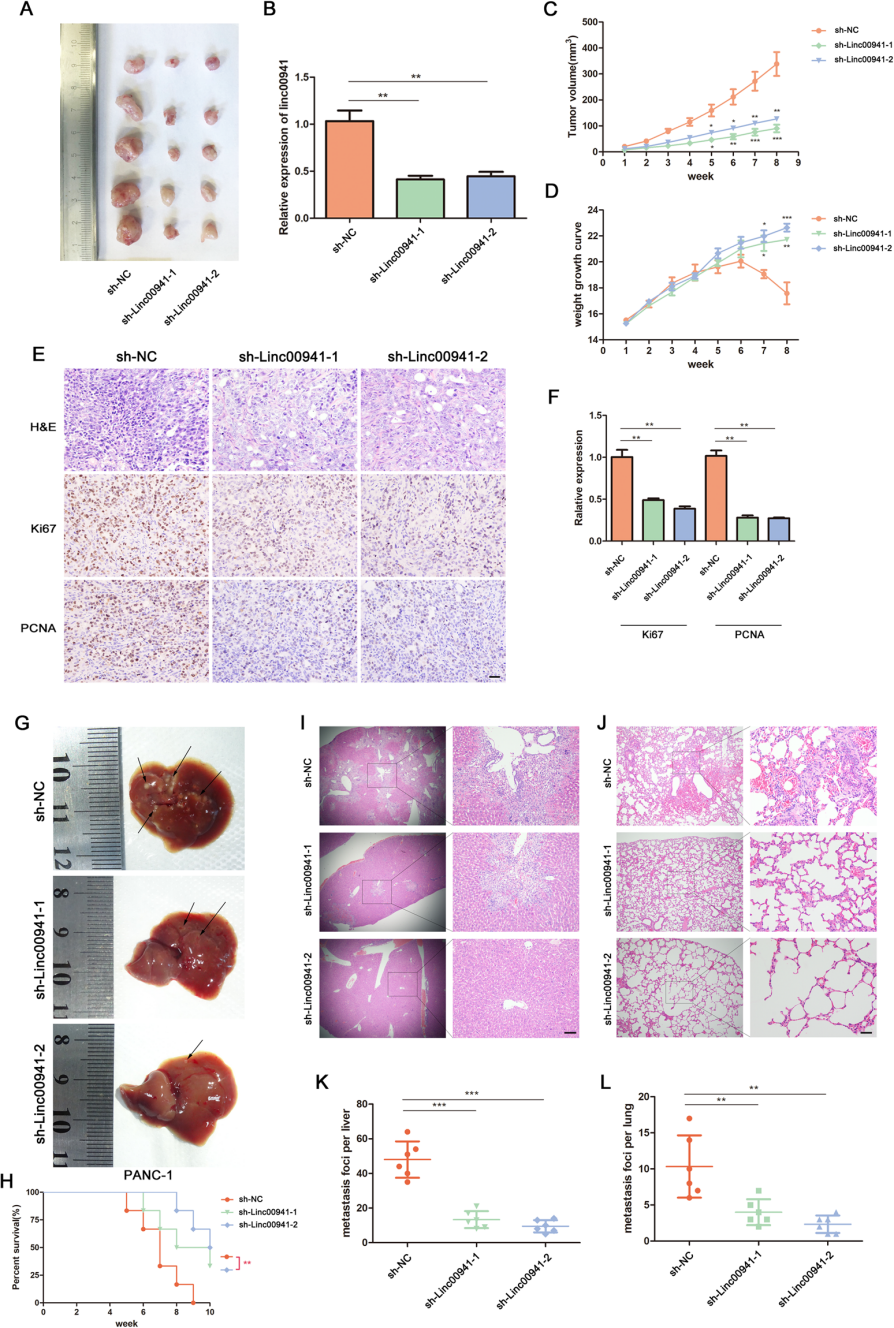
Suppression of LINC00941 expression inhibits PC cell proliferation and metastasis in vivo. **A** Tumor photographs of the subcutaneous xenografts in sh-NC and sh-Linc00941 groups, *n* = 5. **B** The relative RNA expression of LINC00941 in subcutaneous tumors was detected by PCR. **C** Tumor volume of the subcutaneous xenografts in sh-NC and sh-Linc00941. **D** Weight change curve. **E** IHC staining for LINC00941 and representative images of three pairs of subcutaneous xenograft tissue (100×; bar: 100 μm). **F** The relative expression of Ki67 and PCNA in tumor tissue. **G** The PANC-1 cells were conducted the metastasis model for 10 weeks, and the liver metastasis photographs indicated the metastasis loci, *n* = 6 (the arrow shows the metastasis). **H** Survival curve showed the prognosis of mice in sh-NC and sh-Linc00941 groups. Serial sections of whole liver (**I**) and lung (**J**) were H&E stained (bar: 50 μm). Liver (**K**) and lung (**L**) micrometastases were counted. *p < 0.05, **p < 0.01, ***p < 0.001.

To further investigate the role of LINC00941 in PC tumorigeneses and metastasis, we developed an in vivo subcutaneous tumorigenesis model and an in vivo tail vein injection metastatic tumor model, using PANC-1 cells transduced with lentivirus. The findings from the subcutaneous tumorigenesis model suggested that suppression of LINC00941 expression resulted in smaller tumor size and a slower rate of tumor growth (Fig. 3A, C). RNA extracted from harvested tumors was analyzed by RT-qPCR to confirm changes in LINC00941 expression in these tissues. The PCR analysis revealed that LINC00941 expression was decreased in the sh-LINC00941 groups, when compared with the control group (Fig. 3B). In addition, the mice in the control group began to lose weight after 6 weeks of feeding, possibly because they were more prone to cachexia than the mice of the LINC00941 suppression groups (Fig. 3D). A proliferation-related index, based on Ki67 and PCNA immunohistochemistry (IHC) staining, was used to evaluate the tumors of mice. Tissue staining revealed that Ki67 and PCNA were more highly expressed in the control groups than in the LINC00941 silencing groups (Fig. 3E, F). In addition, suppression of LINC00941 expression resulted in significantly fewer liver and lung metastases, and a significantly improved prognosis for these animals (Fig. 3G–L).

### LINC00941 regulates cell proliferation and metastasis by competitively binding miR-335-5p

**Fig. 4:**
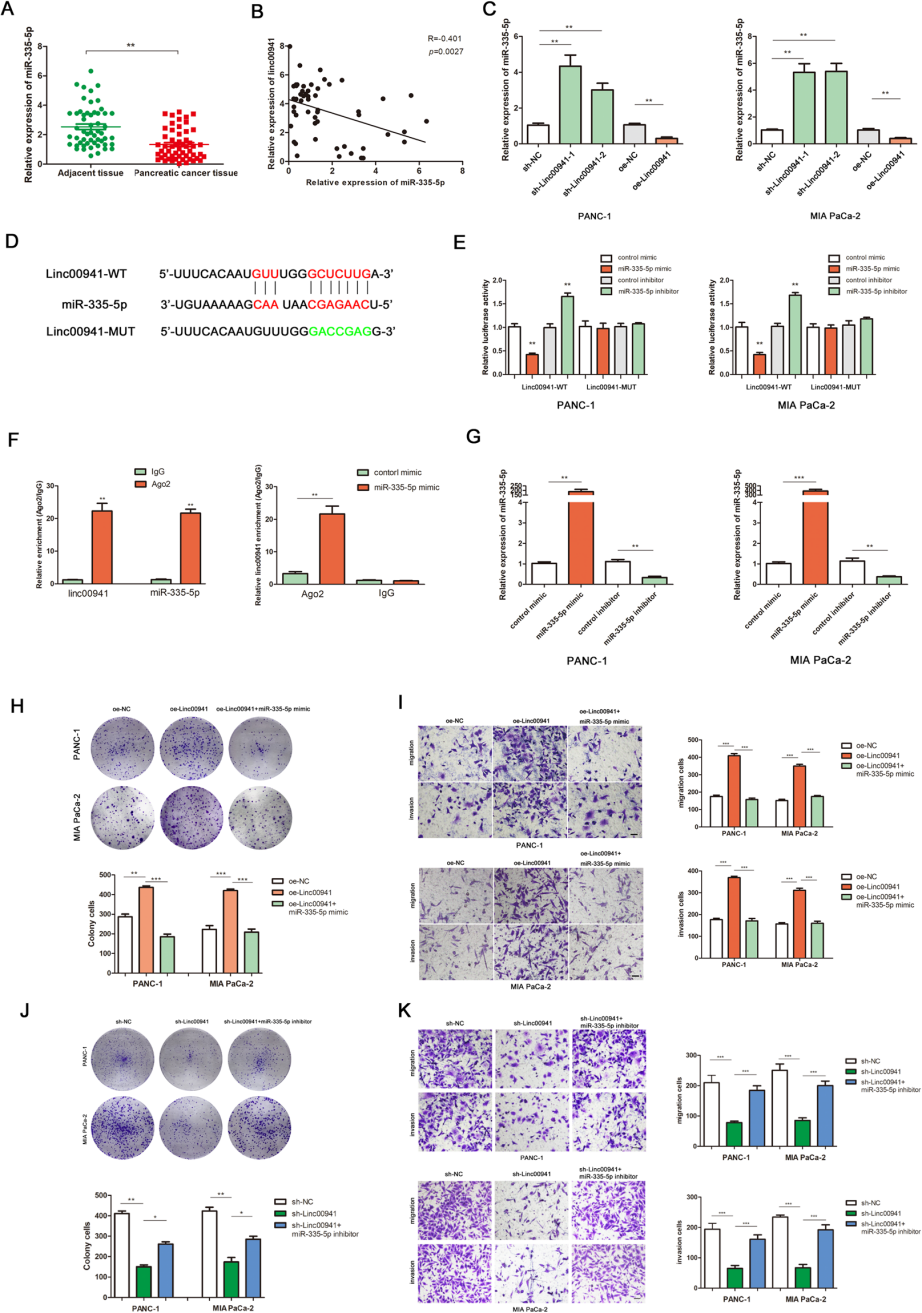
LINC00941 regulated cell proliferation and metastasis by competitively binding to miR-335-5p. **A** RT-qPCR analysis of miR-335-5p expression in PC tissues and cell lines. **B** Pearson correlation analysis was used to investigate the correlation between LINC00941 and miR-335-5p. **C** miR-335-5p expression in sh-NC, sh-Linc00941, oe-NC, and oe-Linc00941 groups. **D** The prediction binding site between miR-335-5p and LINC00941. **E** Luciferase assay was applied to investigate the direct interaction between LINC00941 and miR-335-5p. **F** RIP analysis demonstrated the co-immunoprecipitation of LINC00941 and miR-335-5p. RIP assays were performed in PC cells transfected with or without miR-335-5p mimic. The relative expression was examined by using qRT-PCR analysis. **G** The expression of miR-335-5p was detected by PCR after transfecting miR-335-5p mimics or inhibitors in PC cells. **H**, **I** Functional rescue experiments were performed to verify the effect of miR-335-5p mimic on migration and proliferation in oe-Linc00941 group. **J**, **K** Functional rescue experiments were performed to verify the effect of miR-335-5p inhibitor on migration and proliferation in sh-Linc00941 group. *p < 0.05, **p < 0.01, ***p < 0.001.

To investigate whether LINC00941 could potentially function as a ceRNA for miRNA, we performed a bioinformatics analysis to identify potential targets of LINC00941 using the Starbase3.0 (http://starbase.sysu.edu.cn), seedVicious (https://seedvicious.essex.ac.uk), and LncBase (http://carolina.imis.athena-innovation.gr/diana_tools/web/) databases (Supplemental Fig. 3A, B). miR-335-5p was predicted to interact with LINC00941 in all three databases, and was therefore selected as our candidate miRNA for subsequent experiments. PCR analysis revealed that miR-335-5p expression in PC tissues was decreased relative to adjacent normal tissues (Fig. 4A). Pearson correlation curve analysis suggested a negative correction between LINC00941 and miR-335-5p expression (Fig. 4B). PCR analysis revealed that miR-335-5p expression was negatively associated with LINC00941 expression in PC cells (Fig. 4C). The predicted binding site for miR-335-5p and LINC00941 is displayed in Fig. 4D. A luciferase reporter assay verified this interaction; PC cells expressing the WT LINC00941 reporter demonstrated lower luciferase activity in the miR-335-5p overexpression group and higher luciferase activity in miR-335-5p inhibition group, while the luciferase activity in cells expressing the MUT LINC00941 reporter remained unchanged for either of the miR-335-5p perturbation groups (Fig. 4E). RIP assays further confirmed the direct interaction between LINC00941 and miR-335-5p, which were enhanced in the Ago2 complex (Fig. 4F). PCR analysis confirmed the effects of miR-335-5p mimic or inhibitor transfection on cellular miR-335-5p expression levels (Fig. 4G). Cell function-based rescue experiments revealed that upregulated miR-335-5p could partly antagonize the ability of LINC00941 overexpression to promote the migration, invasion, and proliferation of PC cells (Fig. 4H, I). Further, the miR-335-5p inhibitor was used to transfect with the LINC00941 knockdown PC cells, the results indicated that miR-335-5p could partly reverse the inhibition effect of proliferation and metastasis (Fig. 4J, K).

### MiR-335-5p suppresses cell proliferation, invasion, and migration by regulating ROCK1 expression

**Fig. 5:**
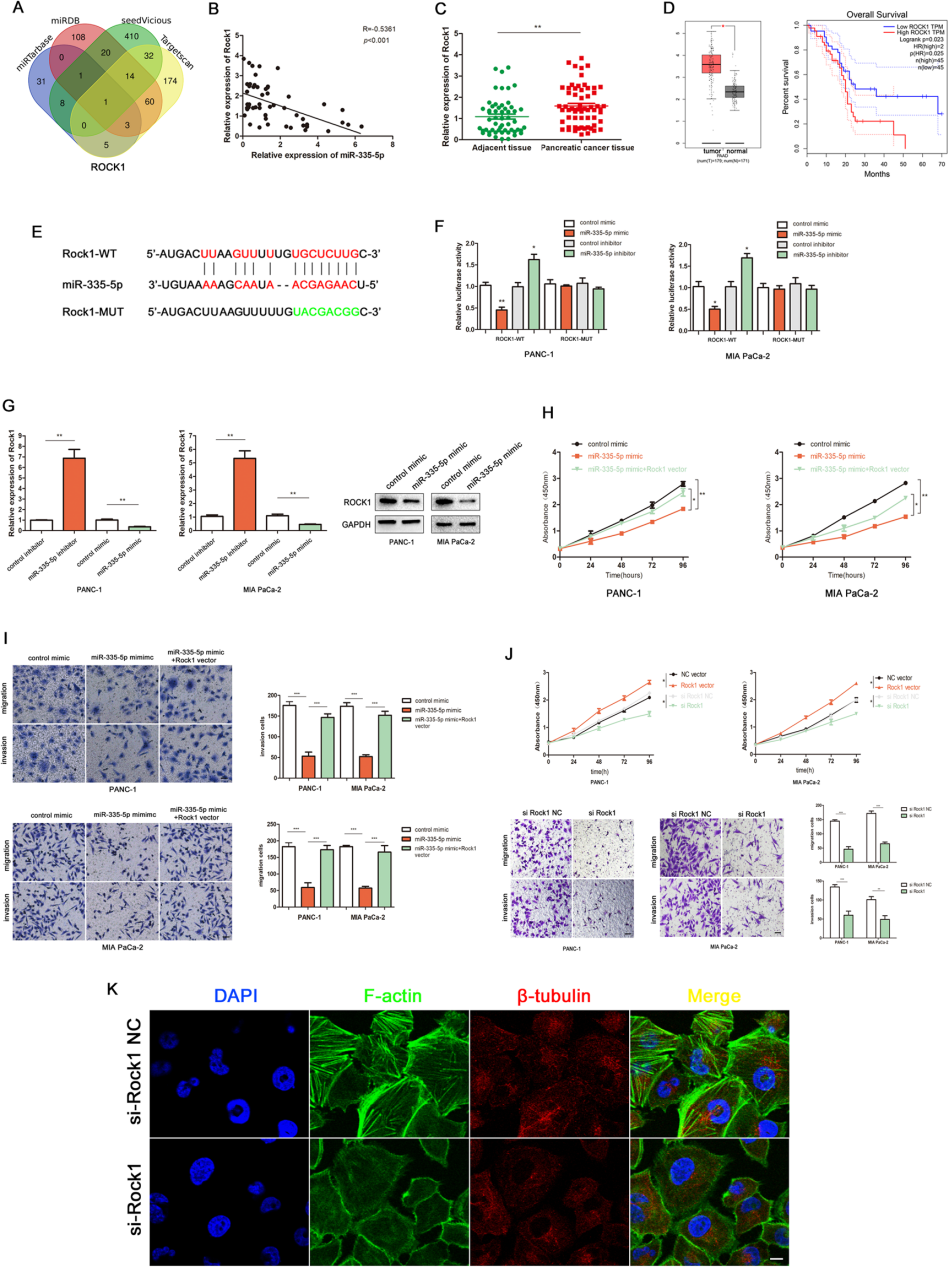
MiR-335-5p suppressed cell proliferation, invasion, and migration through regulating ROCK1 expression. **A** ROCK1 was identified as the target of miR-335-5p from 867 potential targets by Venn analysis. **B** Pearson correlation analysis was used to investigate the correlation between ROCK1 and miR-335-5p. **C** The relative ROCK1 expression in PC tissues. **D** The expression and overall survival curve of ROCK1 in PC from TCGA database. **E** The prediction binding site of miR-335-5p in ROCK1 3′-UTR. **F** Luciferase assay was applied to investigate the direct interaction between ROCK1 and miR-335-5p. **G** The mRNA and protein expression of ROCK1 were detected by PCR after transfecting miR-335-5p mimics or inhibitors in PC cells. **H**, **I** Functional rescue experiments were performed to verify the effect of ROCK1 on migration and proliferation in miR-335-5p group. **J** Functional experiments showed the migrated and growth ability of ROCK1 overexpression on PC cells. **K** Immunofluorescence showed the cytoskeleton of PANC-1 in si-ROCK1 NC and si-ROCK1 groups. *p < 0.05, **p < 0.01, ***p < 0.001.

To further investigate the function of the miRNA target of LINC00941, we performed a bioinformatics analysis to search for target mRNAs of miR-335-5p using the Targetscan (http://www.targetscan.org/vert_72/), miRDB (http://www.mirdb.org/), miRTarbase (http://mirtarbase.cuhk.edu.cn/php/index.php), and Seedvicious (https://seedvicious.essex.ac.uk/) databases. ROCK1 was identified as the target of miR-335-5p from 867 potential targets by Venn analysis (Fig. 5A). In addition, Pearson correlation curve analysis showed a negative correction between ROCK1 and miR-335-5p expression (Fig. 5B). Next, we examined the expression of ROCK1 mRNA in PC tumor tissues and verified that ROCK1 was highly expressed in these tissues (Fig. 5C). Also, the data from TCGA database showed that ROCK1 expression was elevated in PC tumor tissues and that ROCK1 overexpression was significantly correlated with poor prognosis (Fig. 5D). The predicted binding site for miR-335-5p within the 3′-UTR of ROCK1 is displayed in Fig. 5E. Luciferase reporter assays confirmed this prediction, showing that miR-335-5p overexpression induced lower luciferase activity in cells expressing the WT ROCK1 reporter, while miR-335-5p knockdown promoted higher luciferase activity from this reporter (Fig. 5F). However, in cells expressing a MUT ROCK1 reporter in which the putative miR-335-5p target site had been abolished, perturbation of miR-335-5p expression had no effect on reporter activity. PCR and western blotting assays confirmed that ROCK1 mRNA and protein expression were negatively correlated with miR-335-5p expression (Fig. 5G). CCK-8 and transwell assays demonstrated that elevated miR-335-5p expression suppressed cell proliferation, invasion, and migration (Fig. 5H, I). However, cell function-based rescue experiments indicated that elevated ROCK1 expression could partly reverse the suppressive effects of highly expressed miR-335-5p on proliferation, invasion, and migration (Fig. 5H, I).

Next, we investigated the role of ROCK1 in PC cells. Functional experiments revealed that silencing of ROCK1 expression significantly inhibited cell proliferation, invasion, and migration (Fig. 5J). Immunofluorescence analysis of cytoskeletal morphology in PANC-1 cells revealed reduced levels of polymerized F-actin in cells of the ROCK1 knockdown group, when compared with the cells of the negative control group (Fig. 5K).

### LINC00941 competes with ROCK1 for miR-335-5p binding to upregulate ROCK1-dependent LIMK1/Cofilin-1 signaling

**Fig. 6:**
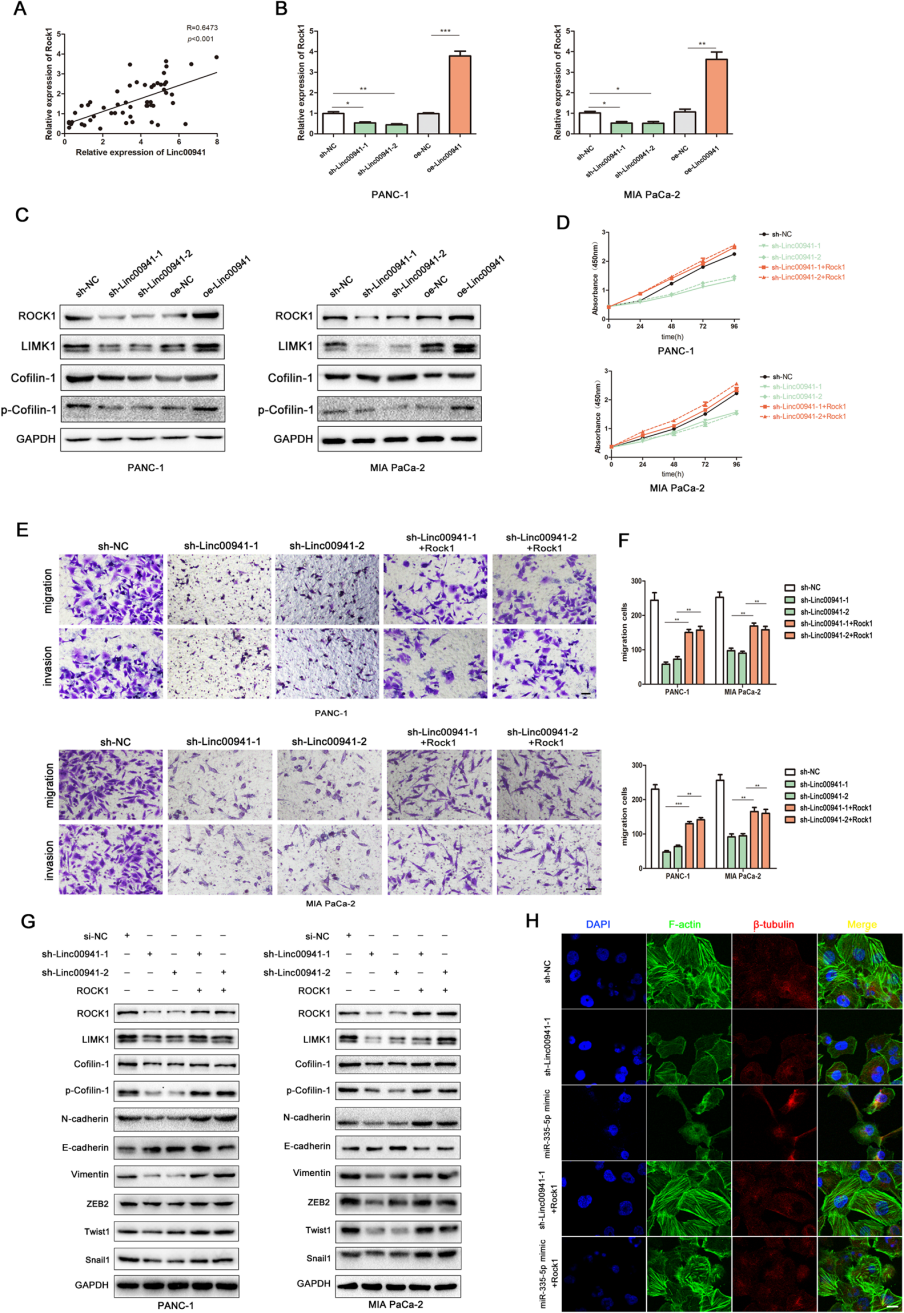
LINC00941 competes with ROCK1 for miR-335-5p binding to upregulate ROCK1-dependent LIMK1/Cofilin-1 signaling. **A** Pearson correlation analysis was used to investigate the correlation between ROCK1 and LINC00941. **B** The mRNA expression of ROCK1 was detected by PCR after transfecting upregulated or downregulated LINC00941 in PC cells. **C** The protein expression of ROCK1, LIMK1, Cofilin-1, and phosphorylated Cofilin-1 after transfecting upregulated or downregulated LINC00941 in PC cells. **D**–**F** Functional rescue experiments were performed to verify the effect of ROCK1 overexpression on migration and proliferation in sh-Linc00941 group. **G** The protein expression changes of ROCK1, LIMK1, Cofilin-1, phosphorylated Cofilin-1, N-cadherin, E-cadherin, Vimentin, ZEB2, Twist1, and Snail1 in sh-Linc00941 + ROCK1 and miR-335-5p mimic + ROCK1 groups compared to sh-Linc00941 and miR-335-5p groups. **H** Immunofluorescence showed the cytoskeleton changes of PANC-1 in sh-Linc00941 + ROCK1 and miR-335-5p mimic + ROCK1 groups compared to sh-Linc00941 and miR-335-5p groups. *p < 0.05, **p < 0.01, ***p < 0.001.

**Fig. 7:**
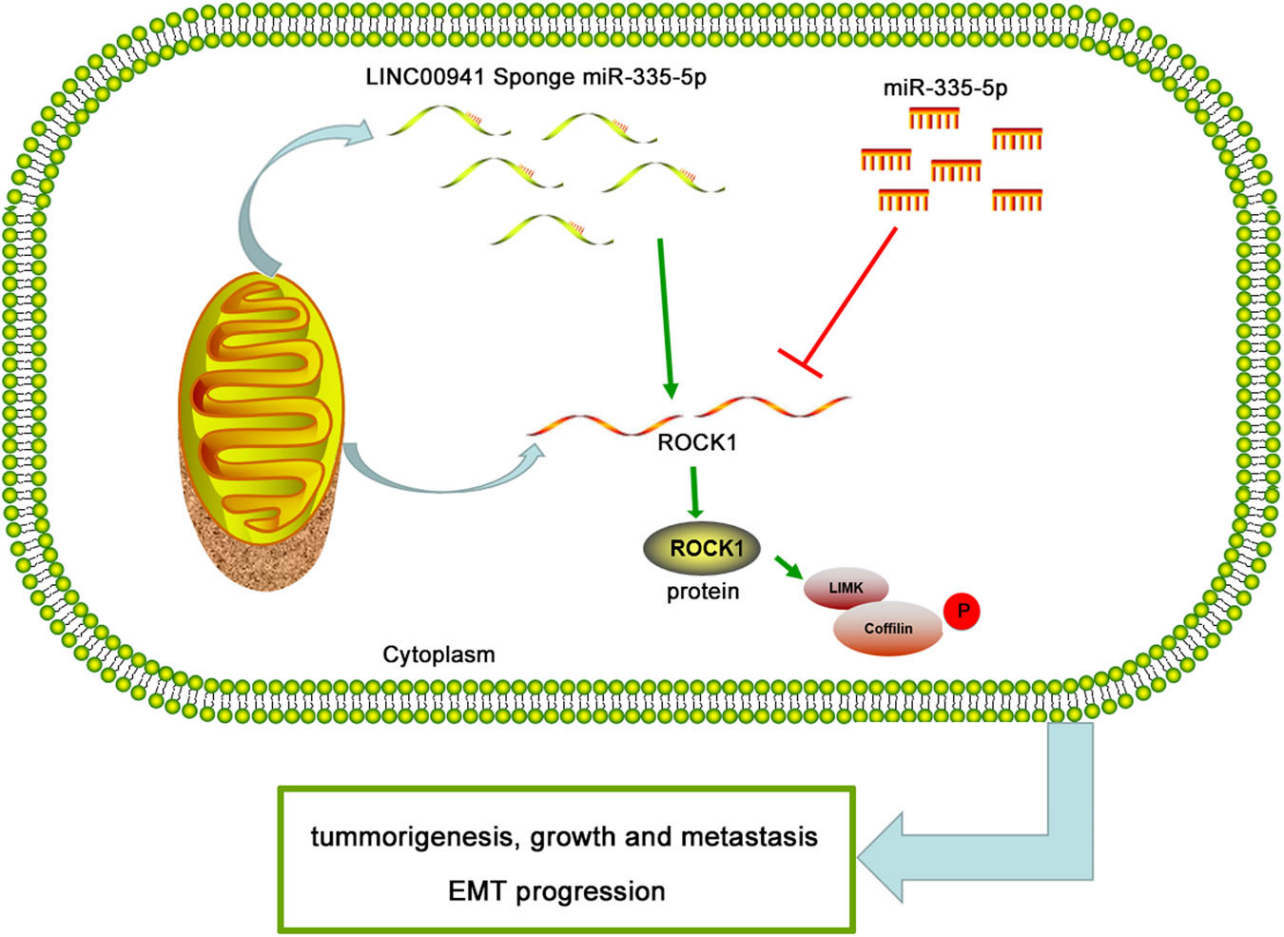
Schematic diagram of mechanism on this study. LINC00941 promotes proliferation, metastasis, and EMT and activates ROCK1-mediated LIMK1/Cofilin-1 pathway via decoying of miR-335-5p in PC cells.

To further establish the underlying mechanism by which LINC00941 facilitated cell growth, metastasis, and EMT, we sought to clarify the connection between LINC00941 and ROCK1. Pearson correlation curve analysis revealed a positive correlation between ROCK1 and LINC00941 expression based on the specimens from our hospital PC patients (Fig. 6A). In addition, the TCGA database result was presented in the (Supplemental Fig. 4). PCR assays further confirmed this relationship, demonstrating that high levels of LINC00941 expression resulted in an upregulation of ROCK1 expression (Fig. 6B). In addition, western blotting assays demonstrated that LINC00941 knockdown significantly decreased protein levels of ROCK1, LIMK1, Cofilin-1, and phosphorylated Cofilin-1, while conversely, LINC00941 overexpression upregulated the expression of these proteins (Fig. 6C). Rescue experiments showed that upregulated ROCK1 expression could partly reverse the inhibitory effects of LINC00941 knockdown on cell proliferation, invasion, and migration (Fig. 6D–F). To verify that the oncogenic functions of LINC00941 were dependent upon its ability to competitively bind miR-335-5p to promote ROCK1-mediated LIMK1/Cofilin-1 pathway activation, rescue experiments were performed and key pathway components then analyzed by western blotting. These experiments revealed that downregulation of LINC00941 or upregulation of miR-335-5p significantly suppressed LIMK1/Cofilin-1 pathway activation, and inhibited the expression of EMT-related proteins (Fig. 6G). However, elevated ROCK1 expression could overcome this inhibition, promoting LIMK1/Cofilin-1 pathway activation and the expression of EMT-related proteins (Fig. 6H). An analysis of cytoskeletal morphology in PANC-1 cells revealed that downregulation of LINC00941 or upregulation miR-335-5p resulted in a dramatic reduction in levels of F-actin, suggesting a state favoring actin depolymerization. Conversely, upregulation of ROCK1 expression resulted in higher levels of F-actin in these cells, suggesting a shift toward a state favoring actin polymerization (Fig. 6H). Our data suggest that LINC00941 plays an important role in promoting PC proliferation, invasion, and metastasis by competitively interacting with miR-335-5p involving in ROCK1-mediated LIMK1/Cofilin-1 pathway (Fig. 7). These findings highlight the LINC00941/miR-335-5p/ROCK1 axis as a potential target for both therapeutic intervention and the development of diagnostic biomarkers for the treatment and management of PC.

## Discussion

PC is a common and aggressive malignancy of the digestive system that has an enormous impact on public health worldwide^14^. Given the limitations of current diagnostic and treatment methods for PC, it is vital that we develop effective new strategies to better combat this disease. There is accumulating evidence to suggest that the dysregulation of lncRNA plays an essential role in the regulation of PC progression. A study by Guo et al. revealed that overexpression of the lncRNA SNHG16 significantly enhanced the migration and invasive potential of PC cells through its ability to sponge miR-200a-3p (ref. ^15^). In addition, a study be Shi et al.^17^ suggested that LINC00346 interacts with miR-188-3p to promote PC cell proliferation and resistance to gemcitabine. The authors went on to show that the underlying molecular mechanism driving these cellular changes could attribute the ability of LINC00346 to competitively sponge miR-188-3p and thereby promote an increase in BRD4, a protein implicated in the cell proliferation and gemcitabine chemoresistance of PC^16,17^. However, some lncRNAs, such as LINC00673, have been reported to function as tumor suppressors in PC. LINC00673 expression was found to be significantly reduced in PC cells, where it functions to suppress cell invasion and migration by inhibiting miR-504 (ref. ^18^). In addition, the lncRNA PXN-AS1 was identified as a ceRNA for miR-3064 and, through miR-3064 sequestration, was found to promote the expression of the tumor suppressor PIP4K2B, resulting in the inhibition on PC progression^19^. LINC00941 was first identified in gastric cancer, where its elevated expression was associated with invasion depth, lymphatic metastasis, and TNM stage. Moreover, enrichment analysis revealed that LINC00941 was significantly correlated with biological processes associated with tumor progression, such as the cell cycle, migration, cell division, and the immune system^8,20^. It has also been suggested that LINC00941 plays an important role in poorly differentiated keratinocytes within the human epidermis, where it inhibits early differentiation by regulating the abundance of SPRR5 (ref. ^21^). By building a co-expression network and exploring functional annotations, Hu and colleagues found LINC00941 gene function to be related to focal adhesion, ECM–receptor interaction, pathways in cancer, and cytokine–cytokine receptor interaction terms, and identified LINC00941 as an optimal diagnostic lncRNA for head and neck squamous cell carcinoma^22^. In our study, we have identified, for the first time, an oncogenic role for LINC00941 in PC. LINC00941 was highly expressed in PC tissues and cells, and elevated LINC00941 expression positively correlated with tumor size, lymphatic metastasis, and poor prognosis. Furthermore, silencing of LINC00941 expression resulted in the inhibition of tumor cell proliferation and metastasis, both in vivo and in vitro.

Aberrant miRNA expression is closely associated with tumorigenesis and progression in a wide variety of cancers^23^. Using a bioinformatics approach, we identified miR-335-5p as a potential target of LINC00941, suggesting that LINC00941 may function as a ceRNA. Notably, miR-335-5p expression was low in PC tissues and when overexpressed, miR-335-5p significantly suppressed cell growth and metastasis. A number of studies have shown that miR-335-5p predominantly acts as a tumor suppressor in cancer, and loss of miR-335-5p expression may therefore be important for tumor development and progression. Zhang et al. have shown that miR-335-5p antagonizes LDHB expression to inhibit cell growth and migration in colorectal cancer^24^. By directly binding to the 3′-UTR of BCL2L2 and inhibiting its expression, miR-335-5p has been shown to restored cisplatin sensitivity in ovarian cancer cells^25^. Dysregulation of miR-335-5p in PC cells has also been found to inhibit cell proliferation by suppressing c-met expression^26^.

ROCK1 has also been associated with proliferation and metastasis in various cancers^27–30^, and ROCK1 mRNA is a known target for many miRNAs^27–29^. Activation of ROCK signaling has been shown to promote extracellular matrix remodeling during PC progression, and it has been suggested that ROCK inhibitors may have utility in suppressing the invasive growth of PC cells by impairing stromal collagen remodeling^27–29^. Previous reports have shown that the ROCK1/LIMK1/Cofilin-1 pathway can influence the growth and migration of tumor cells via regulating actin cytoskeletal dynamics^31^. LIMK1 is a downstream target of ROCK1, and plays an important role in the regulation of the actin cytoskeleton by phosphorylating cofilin on serine 3 (ref. ^32^). Cofilin-1 is the principal substrate of LIMK1 and has the ability to depolymerize F-actin when in its unphosphorylated, active state^32,33^. ROCK1-mediated LIMK1 activation result in cofilin-1 phosphorylation and inactivation, resulting in the inhibition of its actin-severing activity, which is beneficial for F-actin stabilization^32^. In our study, we identified ROCK1 as a target of miR-335-5p and showed that ROCK1 expression is positively correlated with that of LINC00941 in PC. We further demonstrated that LINC00941 facilitates PC progression by competitively binding miR-335-5p to activate ROCK1-mediated LIMK1/cofilin-1 pathway activation.

In summary, we identified an oncogenic role for LINC00941 in the proliferation and metastasis of PC. In addition, we have shown that LINC00941 can function as a ceRNA and can compete for miR-335-5p binding to promote ROCK1 signaling. Our data suggest a mechanism whereby LINC00941-dependent sequestration of miR-335-5p suppresses miR-335-5p-dependent downregulation of ROCK1. The resulting elevation in ROCK1 expression drives LIMK1/Cofilin-1 signaling that promotes pro-tumor cellular behaviors. LINC00941 may therefore have potential utility as a diagnostic biomarker and treatment target in PC.

## Supplementary information

Supplementary figure and table legends

supplemental table1

supplemental Figure1

supplemental Figure2

supplemental Figure3

supplemental Figure4

## Data Availability

All data generated and analyzed during this study are included in this published article are available on request.
