# Supplementary material for: Long noncoding RNA LINC00941 promotes pancreatic cancer progression by competitively binding miR-335-5p to regulate ROCK1-mediated LIMK1/Cofilin-1 signaling
Source: Cell Death Dis. 2021 Jan 4;12(1):36. doi: 10.1038/s41419-020-03316-w (PMC7791140; doi:10.1038/s41419-020-03316-w)
Supplement: Supplementary file 1 — Supplementary figure and table legends [file 41419_2020_3316_MOESM1_ESM.docx]

**Supplemental table 1. The primer sequences in this study.**

**Supplemental Figure 1. LINC00941 was highly expressed in PC tissues and cells and predicted poor prognosis.** (a) The expression level of LINC00941 in GEO database (GSE63124). (b) ROC curve. (c) Specific parameters in the ROC curve.

**Supplemental Figure 2.**  **The mRNA expression of N-cadherin, E-cadherin, Vimentin, ZEB2, Twist1 and Snail1 were detected in up- and down-regulated LINC00941 groups by qRT-PCR**. (a) PANC-1 cell line. (b) MIA PaCa-2 cell line. **p* < 0.05, ***p* < 0.01, ****p* < 0.001

**Supplemental Figure 3. Bioinformation prediction soft analysis of the miRNAs of LINC00941.** (a-c) The prediction of lncRNA and miRNA binding sites in the websites of Starbase3.0 (http://starbase.sysu.edu.cn), seedVicious (https://seedvicious.essex.ac.uk) and LncBase (http://carolina.imis.athena-innovation.gr/diana_tools/web/) databases.

**Supplemental Figure 4. Bioinformation analysis the correlation of LINC00941 and ROCK1 expression in the TCGA database.**
