# Supplementary material for: Long noncoding RNA LINC00941 promotes pancreatic cancer progression by competitively binding miR-335-5p to regulate ROCK1-mediated LIMK1/Cofilin-1 signaling
Source: Cell Death Dis. 2021 Jan 4;12(1):36. doi: 10.1038/s41419-020-03316-w (PMC7791140; doi:10.1038/s41419-020-03316-w)
Supplement: Supplementary file 2 — supplemental table1 [file 41419_2020_3316_MOESM2_ESM.docx]

| Gene | Sequence | |
| --- | --- | --- |
| GAPDH | Forward | 5′-GGAGCGAGATCCCTCCAAAAT-3′ |
|  | Reverse | 5′-GGCTGTTGTCATACTTCTCATGG-3′ |
| U6 | Forward | 5′-TGCGGGTGCTCGCTTCGGC-3′ |
|  | Reverse | 5′-CCAGTGCAGGGTCCGAGGT-3′ |
| LINC00941 | Forward | 5′-GACCTTTTCAGGCCAGCATT-3′ |
|  | Reverse | 5′-ACAATCTGGATAGAGGGCTCA -3′ |
| miR-335-5p | Forward | 5′-GGGTCAAGAGCAATAACGAA-3′ |
|  | Reverse | 5′-CAGTGCGTGTCGTGGAGT-3′ |
| ROCK1 | Forward | 5′-AGGAAGGCGGACATATTAGTCCCT-3′ |
|  | Reverse | 5′-AGACGATAGTTGGGTCCCGGC-3′ |

**Supplemental table 1. The primer sequences in this study.**
