# Supplementary figures and images for: Long noncoding RNA LINC00941 promotes pancreatic cancer progression by competitively binding miR-335-5p to regulate ROCK1-mediated LIMK1/Cofilin-1 signaling
Source: Cell Death Dis. 2021 Jan 4;12(1):36. doi: 10.1038/s41419-020-03316-w (PMC7791140; doi:10.1038/s41419-020-03316-w)

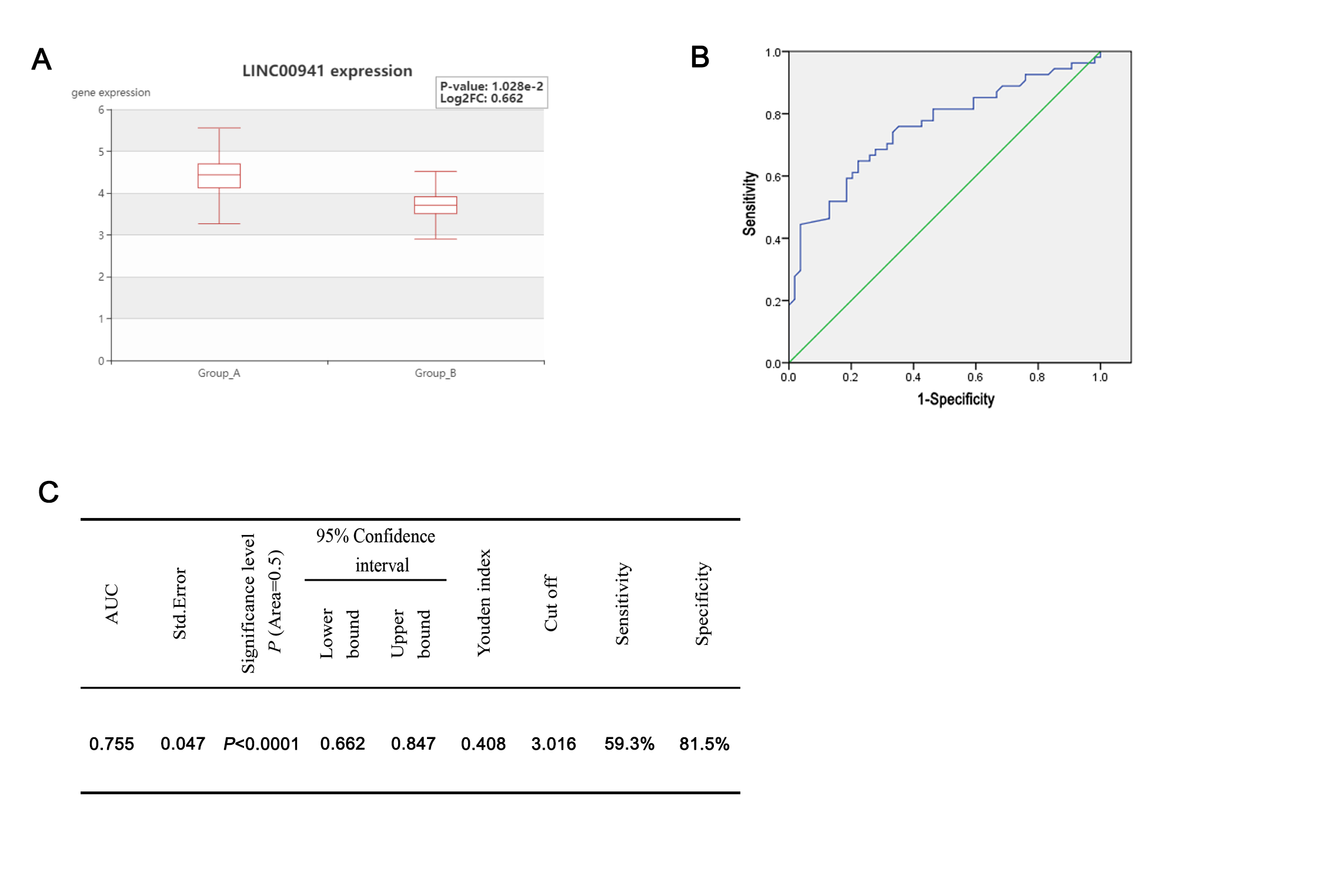

Supplement: Supplementary file 3 — supplemental Figure1 [file 41419_2020_3316_MOESM3_ESM.tif]

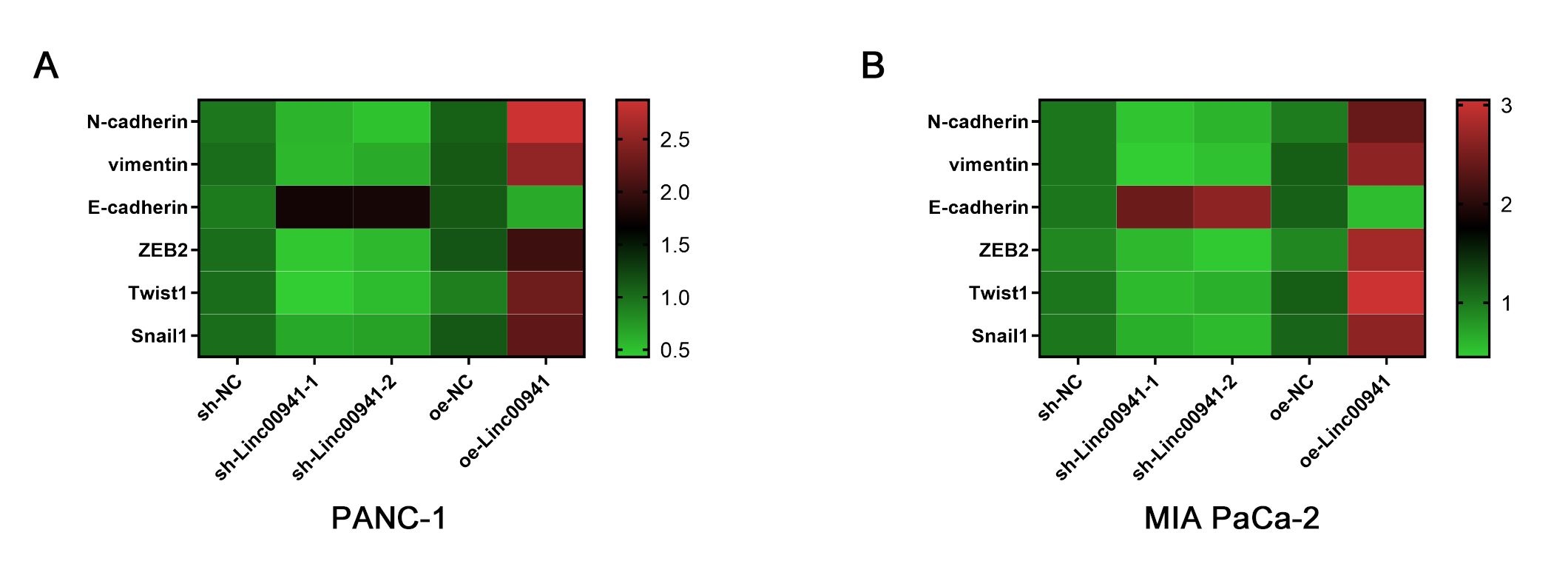

Supplement: Supplementary file 4 — supplemental Figure2 [file 41419_2020_3316_MOESM4_ESM.tif]

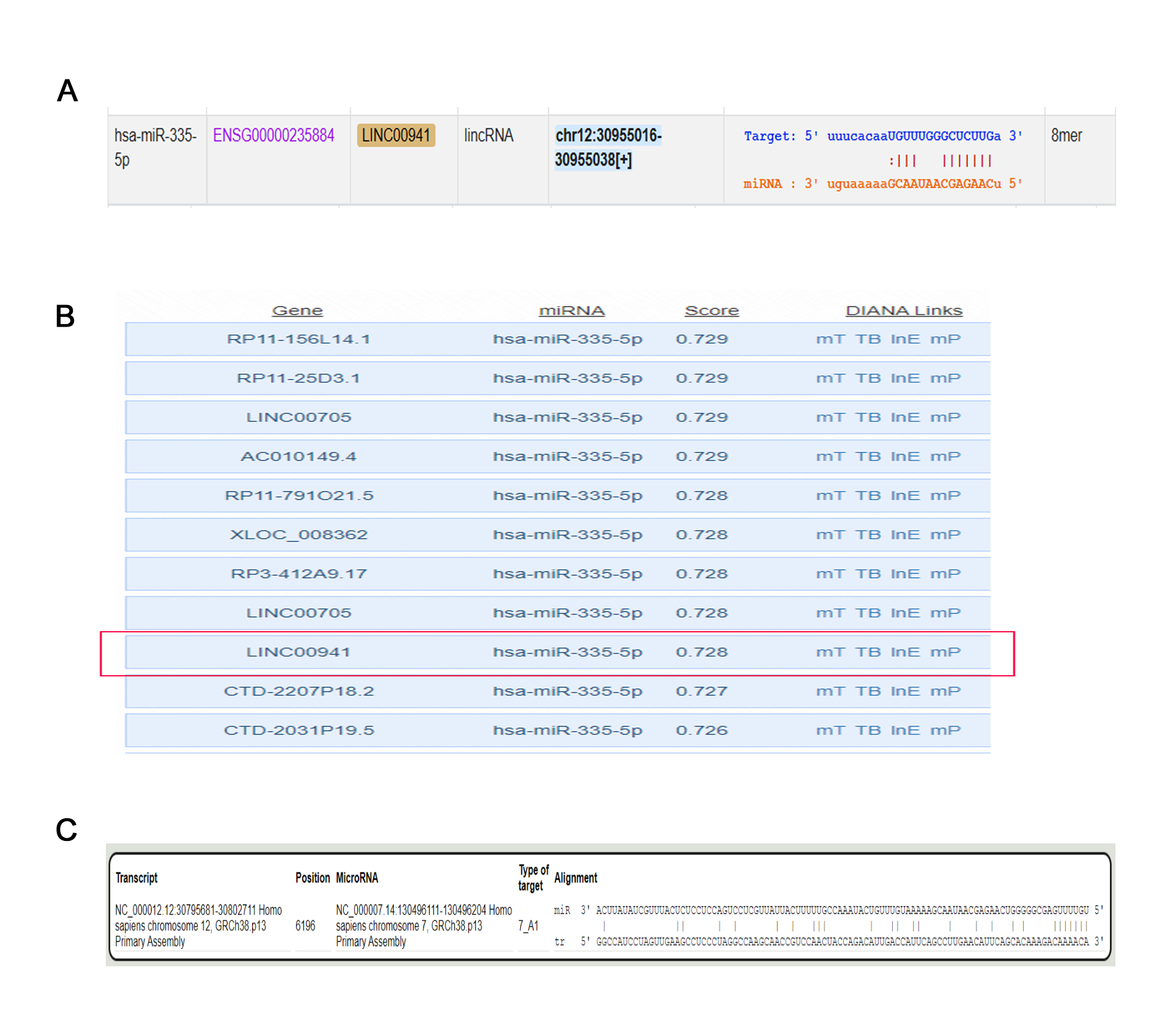

Supplement: Supplementary file 5 — supplemental Figure3 [file 41419_2020_3316_MOESM5_ESM.tif]

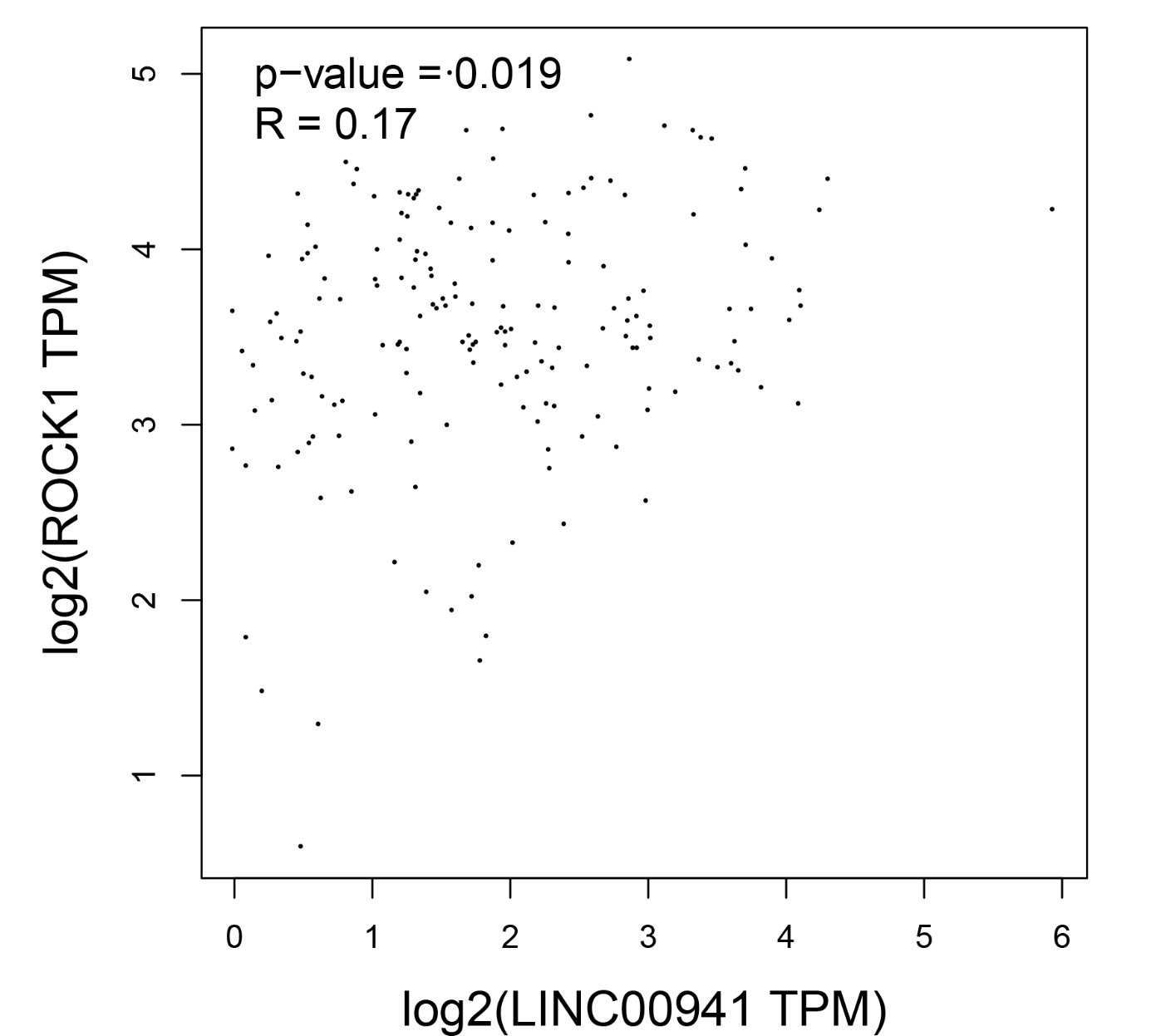

Supplement: Supplementary file 6 — supplemental Figure4 [file 41419_2020_3316_MOESM6_ESM.tif]
